# Impact of socioeconomic status on cancer staging, survival in non-small cell lung cancer

**DOI:** 10.3389/fpubh.2022.992944

**Published:** 2022-11-08

**Authors:** Xianghui Yang, Liyong Deng, Min Li, Yongjie Zhou, Guihua Wang

**Affiliations:** ^1^Department of Oncology, The Affiliated Changsha Central Hospital, Hengyang Medical School, University of South China, Changsha, China; ^2^Interventional Treatment Room, The Affiliated Changsha Central Hospital, Hengyang Medical School, University of South China, Changsha, China; ^3^Department of Interventional Radiology, Zhongshan Hospital, Fudan University, Shanghai, China

**Keywords:** TNM stage, non-small cell lung cancer, SEER database, socioeconomic status, prognosis

## Abstract

**Purpose:**

We performed this study to evaluate the association of socioeconomic status (SES) factors with cancer-specific survival (CSS) of patients with non-small cell lung cancer (NSCLC). We further assessed the predictive value of a novel Tumor Node Metastasis (TNM)-SES staging system, combining the TNM stage with the SES stage.

**Methods:**

Using the Surveillance, Epidemiology, and End Results (SEER) database, we selected 40,378 patients diagnosed with NSCLC from 2012 to 2016. Cox regression method and Harrell's concordance index (C-index) were performed to select the SES factors related to CSS and evaluate the predictive ability of the novel TNM-SES stage. We used Kaplan–Meier curves and a log-rank test to conduct a survival analysis.

**Results:**

We identified four SES factors (marriage, insurance, education, and household income) associated with CSS and constructed the SES stage (SES-1 and SES-2). NSCLC patients with SES-2 stage (low SES) was associated with young adult, black race, male, squamous carcinoma, upper lobe site, and advanced stage. SES-2 stage patients were significantly associated with a dismal prognosis of patients with NSCLC, with a 21.0% increased risk (HR = 1.21, 95%CI (1.18–1.24), *p* < *0.001*). The C-index of our novel TNM-SES stage was 0.732 [95% CI (0.728–0.736)], higher than the traditional TNM stage [0.717, 95% CI (0.715–0.719)], indicating superior predictive value.

**Conclusion:**

*Our population-based study indicated that SES was significantly associated with cancer staging and SCC in patients with NSCLC*. Our novel TNM-SES staging system showed a superior predictive value to the traditional TNM stage. The impact of SES on patients with NSCLC should receive more concern in clinical management.

## Introduction

Lung cancer is the most common cancer and the leading cause of cancer-related death, about 85% of which is non-small cell lung cancer (NSCLC) (1). *The most common subtypes of NSCLC were lung adenocarcinoma (LUAD) and lung squamous cell carcinoma (LUSC)* (2). *The prevalent etiology of lung cancer was cigarette smoking, which is estimated to account for 85 to 90%* (3). Despite much progress made in treatment modalities for lung cancer, such as modified resection, radiation, target therapy and immunotherapy, the 5-year survival remains dismal at 17% (3). A series of variates influenced the prognosis of NSCLC, including biological factors and socioeconomic status (SES). Biological factors' impact on patients' survival with NSCLC have been investigated widely, such as tumor size and lymph invasion (4–8). However, the value of SES on prognosis has not received enough attention in the past. Recently, several studies have shown a significant association between SES and the survival of patients with lung cancer and other malignancy (9–12). Patients with low SES are likely to have an advanced stage of disease and dismal prognosis, compared to patients with high SES.

The American Joint Committee on Cancer (AJCC) staging system has been widely used to predict prognosis and design a treatment plan for individual patients with NSCLC (5). However, the *Tumor Node Metastasis (TNM)* staging system only describes the characteristics of an individual's cancer based on the magnitude of the original (primary) tumor as well as on the extent of cancer, including the extent of the tumor (T), the extent of spread to the lymph nodes (N), and the presence of metastasis (M), without the involvement of SES factors. Hence, a novel staging system that incorporates the SES factor into the TNM stage is needed to be used for the prognosis prediction of patients with NSCLC.

*Our study aimed to evaluate the impact of SES on cancer staging and survival in NSCLC*. By using the Surveillance, Epidemiology, and End Results (SEER) database, we assessed the effect of several SES factors on cancer-specific survival (CSS) and identified SES factors associated with CCS to construct the SES stage. *The difference of clinicopathological features was compared between NSCLC patients of two SES stage*. We built a novel TNM-SES stage that combined the traditional TNM stage and SES stage and further evaluated its predictive value.

## Materials and methods

### Database and patient selection

The clinical information of patients with NSCLC was retrieved from the Surveillance, Epidemiology, and End Results (SEER) program (1973–2015), which was maintained by the National Cancer Institute. The SEER program was a population-based cancer registry and covered ~28% of the United States population.

Patients diagnosed with NSCLC between 1 January 2012 and 31 December 2016 from SEER database were included in our study. Patients with NSCLC were selected by the 3rd edition of the International Classification of Diseases for Oncology (ICD-O-3), and *the histological types codes as following: adenocarcinoma (8,140, 8,230–8,260, 8,310–8,333, 8,470–8,481, 8,490, 8,550), squamous carcinoma (8,052, 8,070–8,073, 8,083–8,084) and other types such as large cell carcinoma (8,012–8,014, 8,031)*. The SES characteristics of the patients with NSCLC involved several variates in our study, such as insurance status, marital status, country percentage with a bachelor's degree, country-level median household income, and country percentage of employed. The classification of insurance and marital status were following the information of patients in the SEER program. The quadratic method was performed for the classification of bachelor's degree, employed percentage, and median household income. Patients aged 18–65 years at diagnosis were enrolled in our study, due to patients aged ≥ 65 years being compelled to receive Medicare in the US, which may bring statistical bias to our results. Our study only enrolled patients with NSCLC whose survival time was ≥ 1 month and who have adequate clinical information from the SEER program.

### Statistical analysis

Continuous variables were shown as mean values ± SD and compared by Student's *t*-test. We presented the categorical variables as frequencies and evaluated them by using the chi-square test or Fisher's exact test. The endpoint in our study was cancer-specific survival (CSS), which was the interval from the time of diagnosis until death from NSCLC. We used the cox proportional hazards regression model to analyze the prognostic factor associated with CSS and calculate the hazard ratios (HRs) with their 95% confidence intervals (95% CIs). The Hosmer-Lemeshow test was used to evaluate the fit of the cox regression model.

The prognostic SES factors were identified by a cox regression model. The coefficients of prognostic SES factors were following the corresponding hazard ratios (HRs) values. By adding parameter values of prognostic SES factors, we gained the SES prognostic scores of each patient with NSCLC. Then, the patients with NSCLC in our study were allocated into two cohorts according to the cutoff SES prognostic scores. We divided the patients with SES prognostic scores < cutoff score into the SES-1 stage group and the patients with scores ≥ cutoff score into the SES-2 stage group. Furthermore, we constructed the new TNM-SES stage, which associated the TNM stage with the new SES stage.

The Kaplan–Meier curves and the log-rank test was performed for the survival analysis. The predictive value of the TNM stage and TNM-SES stage was evaluated by using Harrell's concordance index (C-index), and the higher C-index indicated better predictive performance. We used the R software (version 3.5.1) and GraphPad Prism software version 6.0 (GraphPad Software, Inc., La Jolla, CA) to make the statistical analysis. *P* < 0.05 was considered statistically significant.

## Results

### Clinicopathological features of patients with NSCLC

A total of 40,378 patients diagnosed with NSCLC between 1 January 2012 and 31 December 2016 were retrieved from the SEER database in our study. The demographics and characteristics of the eligible patients are illustrated in Table 1. The mean age of patients with NSCLC was 56.54 ± 6.39, with 21,826 (54.1%) males and 18,552 (45.9%) females. The majority of patients with NSCLC were of White ethnicity (74.8%), insured (70.3%) and married (52.8%). The most histology of NSCLC was adenocarcinoma (65.5%), followed by squamous carcinoma (22.9%) and large cell lung cancer (2.3%). The most frequent site of the tumor was located in the upper lobe (56.5%) and right lung (59.0%). Most patients (51.5%) of NSCLC at initial diagnosis were at stage IV of TNM stage. Notably, only 11,903 patients (29.5%) received surgery treatment and 24,432 patients (60.5%) had undertaken chemotherapy.

**Table 1:** The characteristics of patients with non-small cell lung cancer (NSCLC).

**Characteristic**		**No. (%)**
Age		56.54 ± 6.39
Race	White	30,206 (74.8)
	Black	6,684 (16.6)
	Other^*^	3,488 (8.6)
Sex	Female	18,552 (45.9)
	Male	21,826 (54.1)
Histology	Adenocarcinoma	26,438 (65.5)
	Squamous carcinoma	9,260 (22.9)
	Large cell lung cancer	935 (2.3)
	Other lung cancer	3,745 (9.3)
Site	Upper lobe	22,811 (56.5)
	Middle lobe	1,930 (4.8)
	Lower lobe	9,991 (24.7)
	Main bronchus	1,824 (4.5)
	Overlapping	474 (1.2)
	Non–specified	3,348 (8.3)
*Location*	Right	23,833 (59.0)
	Left	16,096 (39.9)
	Bilateral	449 (1.1)
Insurance status	Insured	28,395 (70.3)
	Medicaid	9,199 (22.8)
	Uninsured	2,784 (6.9)
Marital status	Married	21,324 (52.8)
	*single*	10,678 (26.4)
	Divorced	6,312 (15.6)
	Widowed	2,064 (5.1)
Country % with bachelor degree	7.64–22.82%	10,233 (25.3)
	22.83–31.23%	10,097 (25.0)
	31.24%−39.07	10,283 (25.5)
	39.08–57.51%	9,765 (24.2)
Country % with employed	1.29–5.8%	10,437 (25.8)
	5.81–7.06	9,765 (24.2)
	7.07–8.53	10,297 (25.5)
	8.54–17.16	9,879 (24.5)
Country–level median household income^**^	19.26–52.24 K	10,126 (25.1)
	52.25– 61.02 K	11,866 (29.4)
	61.03–74.75 K	8,454 (20.9)
	74.76–110.97 K	9,932 (24.6)
TNM stage	IA	5,109 (12.7)
	IB	2,689 (6.7)
	IIA	1,662 (4.1)
	IIB	1,677 (4.2)
	IIIA	5,513 (13.7)
	IIIB	2,924 (7.2)
	IV	20,804 (51.5)
Surgery	No surgery	28,475 (70.5)
	Surgery	11,903 (29.5)
Chemotherapy		
	N0	15,946 (39.5)
	Yes	24,432 (60.5)

* Indicates American Indian/AK Native, Asian/Pacific Islander, and unknown.

** Shown in US dollars.

TNM stage: Tumor Node Metastasis (TNM).

Finally, 23,945 patients (59.3%) with NSCLC died due to tumor progression. By using the cox regression model, we identified four SES factors (insurance status, marital status, country percentage with bachelor's degree and country-level median household income) associated with CSS of patients with NSCLC. Besides, other important predictors were age, sex, histology, tumor size, tumor location, TNM stage, and surgery and chemotherapy, as shown in Table 2.

**Table 2:** Univariate and multivariate analysis of CSS of patients with NSCLC.

**Variables**	**Univariate analysis**		**Multivariate analysis**	
	**HR (95% CI)**	***P***	**HR (95% CI)**	***P***
Age	1.06 (1.05–1.09)	<0.001^*^	1.06 (1.05–1.08)	<0.001^*^
Sex				
Female	1 (Reference)	1	1 (Reference)	1
Male	1.40 (1.37–1.44)	<0.001^*^	1.23 (1.20–1.27)	<0.001^*^
Race				
White	1 (Reference)	1	1 (Reference)	1
Black	1.16 (1.12–1.20)	<0.001^*^	0.97 (0.94–1.01)	0.105
Other^**^	0.83 (0.79–0.87)	<0.001^*^	0.73 (0.70–0.77)	<0.001^*^
Histology				
Adenocarcinoma	1 (Reference)	1	1 (Reference)	1
Squamous carcinoma	1.22 (1.18–1.26)	<0.001^*^	1.21 (1.17–1.25)	<0.001^*^
Large cell lung cancer	1.55 (1.44–1.48)	<0.001^*^	1.50 (1.38–1.62)	<0.001^*^
Other lung cancer	1.72 (1.65–1.79)	<0.001^*^	1.31 (1.26–1.37)	<0.001^*^
Site				
Upper lobe	1 (Reference)	1	1 (Reference)	1
Middle lobe	0.86 (0.80–0.91)	<0.001^*^	0.96 (0.90–1.03)	0.269
Lower lobe	0.94 (0.91–0.97)	<0.001^*^	1.02 (0.99–1.06)	0.125
Main bronchus	1.69 (1.60–1.78)	<0.001^*^	1.18 (1.12–1.25)	<0.001^*^
Overlapping	1.19 (1.06–1.33)	0.004^*^	1.23 (1.09–1.38)	0.001^*^
Non–specified	1.90 (1.82–1.99)	<0.001^*^	1.14 (1.09–1.19)	<0.001^*^
Laterality				
Right	1 (Reference)	1	1 (Reference)	1
Left	0.99 (0.96–1.01)	0.355	0.99 (0.96–1.02)	0.399
Bilateral	1.99 (1.79–2.21)	<0.001^*^	0.92 (0.82–1.02)	0.124
TNM stage				
IA	1 (Reference)	1	1 (Reference)	1
IB	1.89 (1.68–2.13)	<0.001^*^	2.03 (1.80–2.28)	<0.001^*^
IIA	2.73 (2.41–3.09)	<0.001^*^	3.46 (3.06–3.92)	<0.001^*^
IIB	3.68 (3.27–4.13)	<0.001^*^	4.11 (3.65–4.62)	<0.001^*^
IIIA	6.430 (5.87–7.04)	<0.001^*^	6.08 (5.53–6.70)	<0.001^*^
IIIB	9.970 (9.07–10.060)	<0.001^*^	7.78 (7.02–8.61)	<0.001^*^
IV	17.57 (16.14–19.13)	<0.001^*^	14.07 (12.82–15.43)	<0.001^*^
Insurance status				
Insured	1 (Reference)	1	1 (Reference)	1
Medicaid	1.43 (1.39–1.47)	<0.001^*^	1.18 (1.15–1.22)	<0.001^*^
Uninsured	1.65 (1.58–1.73)	<0.001^*^	1.21 (1.15–1.27)	<0.001^*^
Marital status				
Married	1 (Reference)	1	1 (Reference)	1
*Single*	1.31 (1.27–1.35)	<0.001^*^	1.11 (1.08–1.15)	<0.001^*^
Divorced	1.21 (1.16–1.250)	<0.001^*^	1.11 (1.07–1.15)	<0.001^*^
Widowed	1.13 (1.06–1.19)	<0.001^*^	1.11 (1.04–1.18)	0.001^*^
Country % with bachelor degree				
7.64–22.82%	1 (Reference)	1	1 (Reference)	1
22.83–31.23%	0.93 (089–0.96)	<0.001^*^	0.96 (0.92–1.00)	0.041^*^
31.24%−39.07%	0.86 (0.83–0.90)	<0.001^*^	0.89 (0.85–0.93)	<0.001^*^
39.08–57.51%	0.78 (0.75–0.81)	<0.001^*^	0.85 (0.80–0.90)	<0.001^*^
Country–level median household income^***^				
19.26–45.37 K	1 (Reference)	1	1 (Reference)	1
45.38– 56.20 K	0.93 (0.90–0.96)	<0.001^*^	0.92 (0.89–0.96)	<0.001^*^
56.21–66.40 K	0.82 (0.79–0.860)	<0.001^*^	0.84 (0.80–0.89)	<0.001^*^
66.41–110.97K	0.77 (0.74–0.80)	<0.001^*^	0.75 (0.71–0.78)	<0.001^*^
Country % with employed				
1.29–5.8%	1 (Reference)	1	1 (Reference)	1
5.81–7.06%	0.97 (0.94–1.01)	0.146	0.99 (0.95–1.02)	0.477
7.07–8.53%	0.99 (0.96–1.03)	0.663	0.94 (0.91–0.98)	0.003^*^
8.54–17.16%	1.14 (1.10–1.18)	<0.001^*^	0.98 (0.94–1.02)	0.331
Surgery				
No surgery	1 (Reference)	1	1 (Reference)	1
Surgery	0.14 (0.13–0.14)	<0.001^*^	0.35 (0.33–0.37)	<0.001^*^
Chemotherapy				
No	1 (Reference)	1	1 (Reference)	1
Yes	1.40 (1.36–1.44)	<0.001^*^	0.50 (0.48–0.51)	<0.001^*^

CSS, cancer-specific survival; NSCLC, non-small cell lung cancer; HR, hazard ratio; CI, confidence interval.

* Indicates significance of P < 0.05.

** Indicates American Indian/AK Native, Asian/Pacific Islander and unknown.

*** Shown in US dollars.

### Patients with NSCLC were divorced into two SES-stage according to SES prognostic scores

**Figure 1:**
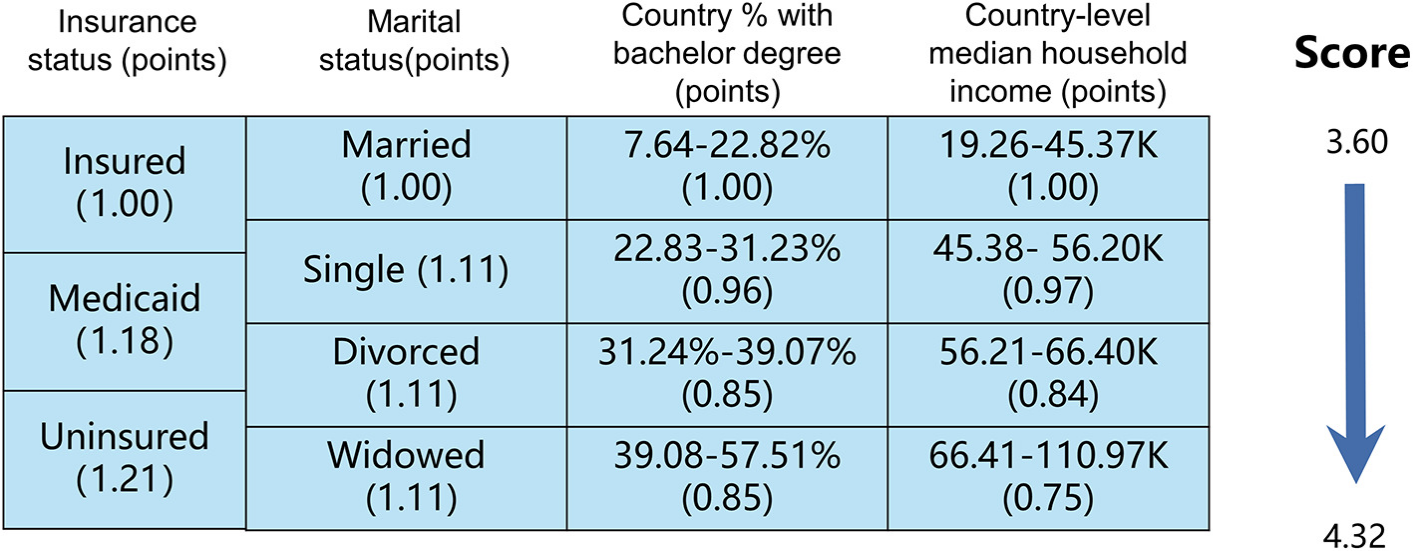
The socioeconomic status (SES) prognostic scores calculation of the patients with NSCLC.

We used the cox regression model to select four SES factors related to cancer-specific survival (CSS), which were insurance status, marital status, country percentage with a bachelor's degree and country-level median household income. *The Hosmer-Lemeshow test showed good calibration (x*^2^ = *5.224, P* = *0.521)*. As illustrated in Figure 1, patients with NSCLC were stratified into subgroups according to the different statuses of the above four SES factors. The SES prognostic scores of each patient were obtained by adding the coefficient of four significant SES factors. In our study, the SES prognostic scores of patients ranged from 3.60 to 4.32, and the score of 4.32 represented the dismal prognosis and the score of 3.60 indicated the optimal prognosis. Then we divided all included patients with NSCLC into two groups based on cutoff SES prognostic score (score of 3.93), 20,237 patients in the SES-1 stage and 20,141 patients in the SES-2 stage. Patients in the SES-1 stage have higher SES status than those in the SES-2 stage. For instance, the uninsured (1.21) and divorced (1.11) patient settled in the country with where 31.24%-39.07% of people with a bachelor's degree (0.89) and the country with 45.38–56.20 K of median household income (0.92), and the SES prognostic score of this patient was 4.13, who was allocated into SES-2 group.

There were significant differences (except tumor location) between the two SES stages, as shown in Table 3. Patients with the SES-1 stage were older than those with the SES-2 stage (56.70 vs. 56.38, *P* < 0.001). Patients with the SES-2 stage were more likely males (56.3% vs. 51.8%, *P* < 0.001) and of Black ethnicity (21.3% vs. 11.9%, *P* < 0.001). The proportion of adenocarcinoma and TNM I stage (IA and IB) was higher in patients with SES-1 stage than that in patients with SES-2 stage (71.2% vs. 59.7%, *P* < 0.001; 21.6% vs. 16.9%, *P* < 0.001). Patients with SES-1 stage were more likely to receive surgery (33.5% vs. 25.4%, *P* < 0.001) and chemotherapy (62.5% vs. 58.5%, *P* < 0.001).

**Table 3:** The characteristics of patients with NSCLC in two SES stage.

**Characteristic**		**SES-1 stage (%)**	**SES-2 stage (%)**	***P***
N		20,237	20,141	
Age		56.70 (6.50)	56.38 (6.27)	<0.001^*^
Race	White	15,084 (74.5)	15,122 (75.1)	<0.001^*^
	Black	2,402 (11.9)	4,282 (21.3)	
	Other^**^	2,751 (13.6)	737 (3.7)	
Sex	Female	9,745 (48.2)	8,807 (43.7)	<0.001^*^
	Male	10,492 (51.8)	11,334 (56.3)	
Histology	Adenocarcinoma	14,404 (71.2)	12,034 (59.7)	<0.001^*^
	Squamous carcinoma	3,728 (18.4)	5,532 (27.5)	
	Large cell lung cancer	401 (2.0)	534 (2.7)	
	Other lung cancer	1,704 (8.4)	2,041 (10.1)	
Site	Upper lobe	11,135 (55.0)	11,676 (58.0)	<0.001^*^
	Middle lobe	1,025 (5.1)	905 (4.5)	
	Lower lobe	5,363 (26.5)	4,628 (23.0)	
	Main bronchus	778 (3.8)	1,046 (5.2)	
	Overlapping	233 (1.2)	241 (1.2)	
	Non-specified	1,703 (8.4)	1,645 (8.2)	
Location	Right	11,955 (59.1)	11,878 (59.0)	0.77
	Left	8,050 (39.8)	8,046 (39.9)	
	Bilateral	232 (1.1)	217 (1.1)	
TNM.stage	IA	2,948 (14.6)	2,161 (10.7)	<0.001^*^
	IB	1,414 (7.0)	1,275 (6.3)	
	IIA	813 (4.0)	849 (4.2)	
	IIB	810 (4.0)	867 (4.3)	
	IIIA	2,672 (13.2)	2,841 (14.1)	
	IIIB	1,347 (6.7)	1,577 (7.8)	
	IV	10,233 (50.6)	10,571 (52.5)	
Surgery	No surgery	13,458 (66.5)	15,017 (74.6)	<0.001^*^
	Surgery	6,779 (33.5)	5,124 (25.4)	
Chemotherapy	0	7,597 (37.5)	8,349 (41.5)	<0.001^*^
	1	12,640 (62.5)	11,792 (58.5)	

NSCLC, non-small cell lung cancer; SES, socioeconomic status.

* Indicates significance of P < 0.05.

** Indicates American Indian/AK Native, Asian/Pacific Islander and unknown.

### Patients with the SES-1 stage had a better prognosis

**Figure 2:**
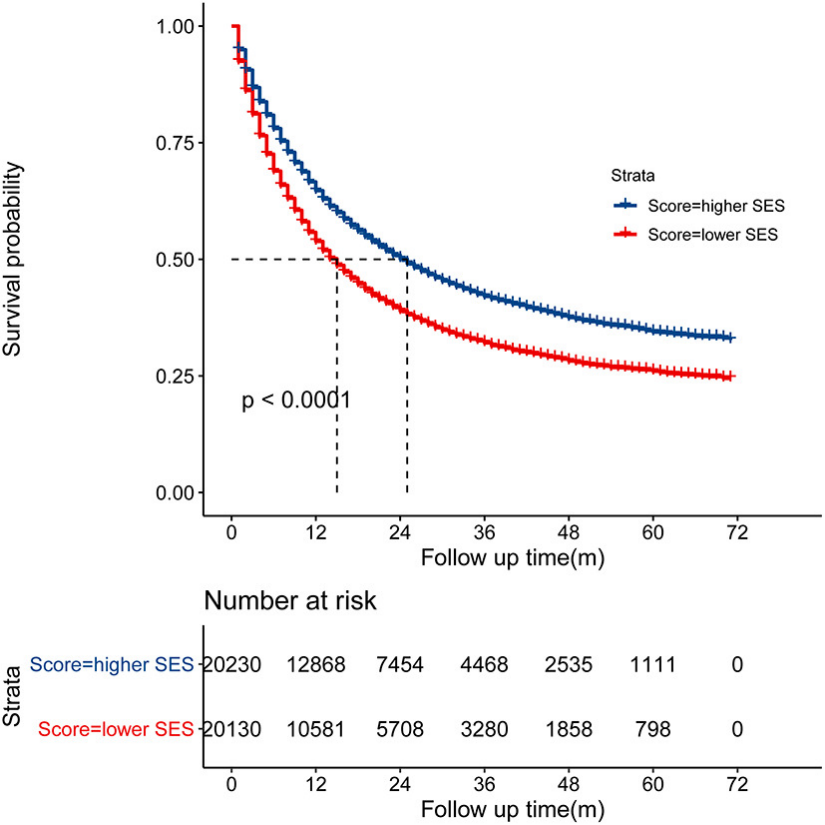
Kaplan-Meier curve showed that the SES-1 stage was associated with better cancer-specific survival (CSS).

In the multivariate analysis, the SES stage was significantly associated with CSS of patients with NSCLC [HR = 1.21, 95% CI (1.18–1.24), *p* < 0.001], as illustrated in Table 4 *(Hosmer-Lemeshow test, x*^2^ = *5.314, P* = *0.537)*. Patients with the SES-1 stage had a better prognosis than patients with the SES-2 stage in the Kaplan-Meier curves (*p* < 0.0001, Figure 2). The same results occurred in the stratified analysis of the association of SES stage and CSS by histology, race, sex and site, as illustrated in Supplementary Figure S1.

**Table 4:** Multivariate analysis of CSS of patients with NSCLC.

**Variables**	**Multivariate analysis**	
	**HR (95% CI)**	***P***
Age	1.01 (1.01–1.01)	<0.001
Sex		
Female	1 (Reference)	1
Male	1.23 (1.19–1.26)	<0.001^*^
Race		
White	1 (Reference)	1
Black	0.99 (0.95–1.02)	0.403
Other^**^	0.73 (0.70–0.77)	<0.001^*^
Histology		
Adenocarcinoma	1 (Reference)	1
Squamous carcinoma	1.22 (1.18–1.26)	<0.001^*^
Large cell lung cancer	1.49 (1.38–1.61)	<0.001^*^
Other lung cancer	1.31 (1.26–1.37)	<0.001^*^
Site		
Upper lobe	1 (Reference)	1
Middle lobe	0.96 (0.90–1.03)	0.262
Lower lobe	1.02 (0.99–1.05)	0.196
Main bronchus	1.19 (1.12–1.25)	<0.001^*^
Overlapping	1.24 (1.10–1.39)	<0.001^*^
Non–specified	1.14 (1.09–1.19)	<0.001^*^
Laterality		
Right	1 (Reference)	1
Left	0.99 (0.96–1.01)	0.307
Bilateral	0.92 (0.83–1.03)	0.142
TNM stage		
IA	1 (Reference)	1
IB	2.03 (1.81–2.29)	<0.001^*^
IIA	3.49 (3.08–3.96)	<0.001^*^
IIB	4.14 (3.69–4.66)	<0.001^*^
IIIA	6.12 (5.56–6.74)	<0.001^*^
IIIB	7.80 (7.05–8.64)	<0.001^*^
IV	14.10 (12.85–15.47)	<0.001^*^
Country % with employed		
1.29–5.8%	1 (Reference)	1
5.81–7.06%	0.99 (0.95–1.02)	0.533
7.07–8.53%	0.94 (0.91–0.98)	0.001^*^
8.54–17.16%	0.97 (0.94–1.01)	0.136
Surgery		
No surgery	1 (Reference)	1
Surgery	0.35 (0.33–0.36)	<0.001^*^
Chemotherapy		
No	1 (Reference)	1
Yes	0.49 (0.48–0.51)	<0.001^*^
SES stage		
SES I	1 (Reference)	1
SES II	1.21 (1.18–1.24)	<0.001^*^

CSS, cancer-specific survival; NSCLC, non-small cell lung cancer; HR, hazard ratio; CI, confidence interval; SES, socioeconomic status.

* Indicates significance of P < 0.05.

** Indicates American Indian/AK Native, Asian/Pacific Islander and unknown.

### The TNM-SES stage had excellent predictive performance

**Figure 3:**
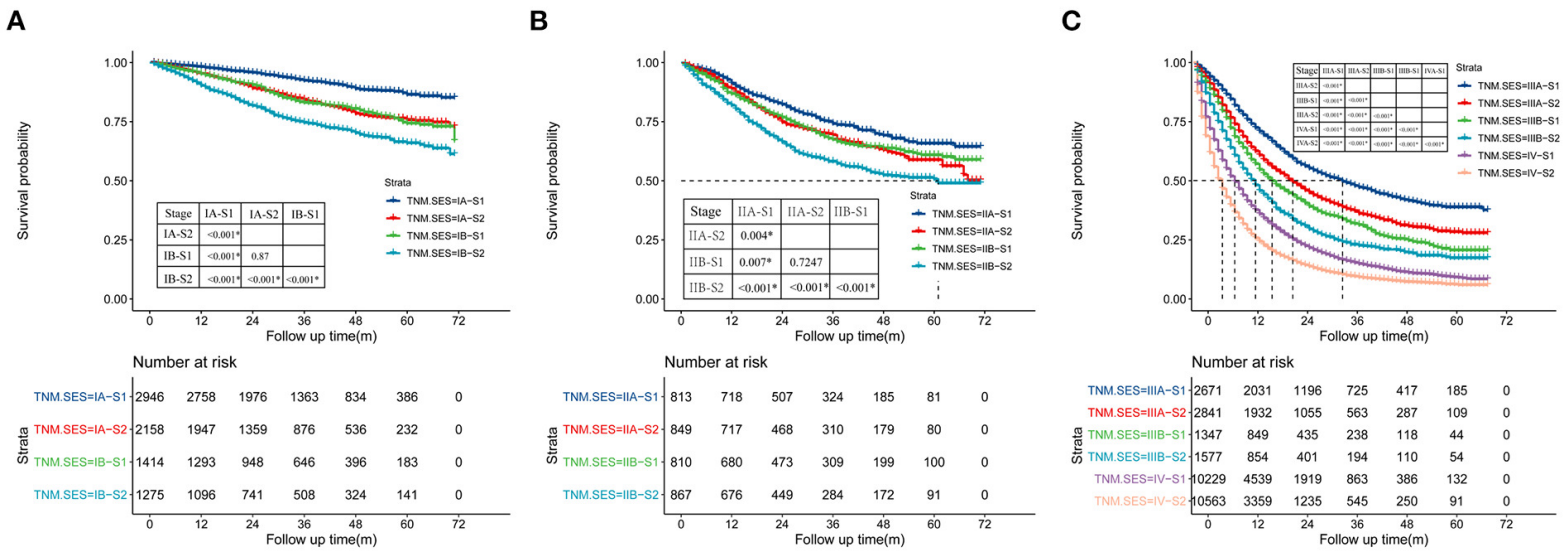
Kaplan-Meier curves of cancer-specific survival (CSS) of the patient with NSCLC in each TNM (tumor node metastasis)-SES (socioeconomic status) stage, and **(A)** for TNM IA, IIB, **(B)** for IIA, IIB, **(C)** for IIIA, IIIB, IV.

The concordance index (C-index) of the TNM-SES stage for predicting CSS was 0.732 [95% CI (0.728–0.736)], which was higher than that of the traditional TNM stage [0.717, 95% CI (0.715–0.719)], showing the excellent predictive performance of the new staging system. The Kaplan–Meier curves showed that SES-1stage patients with NSCLC had a better prognosis than SES-2 stage patients in each TNM stage, with all *P* values < 0.05 (Figure 3). Interestingly, some SES-1 stage patients had a similar prognosis compared to SES-2 stage patients with a higher TNM stage. For instance, there was no significant CSS in IA-S2 stage patients and IB-S1stage patients, also in IIA-S2 stage patients and IIB-S1stage patients.

**Figure 4:**
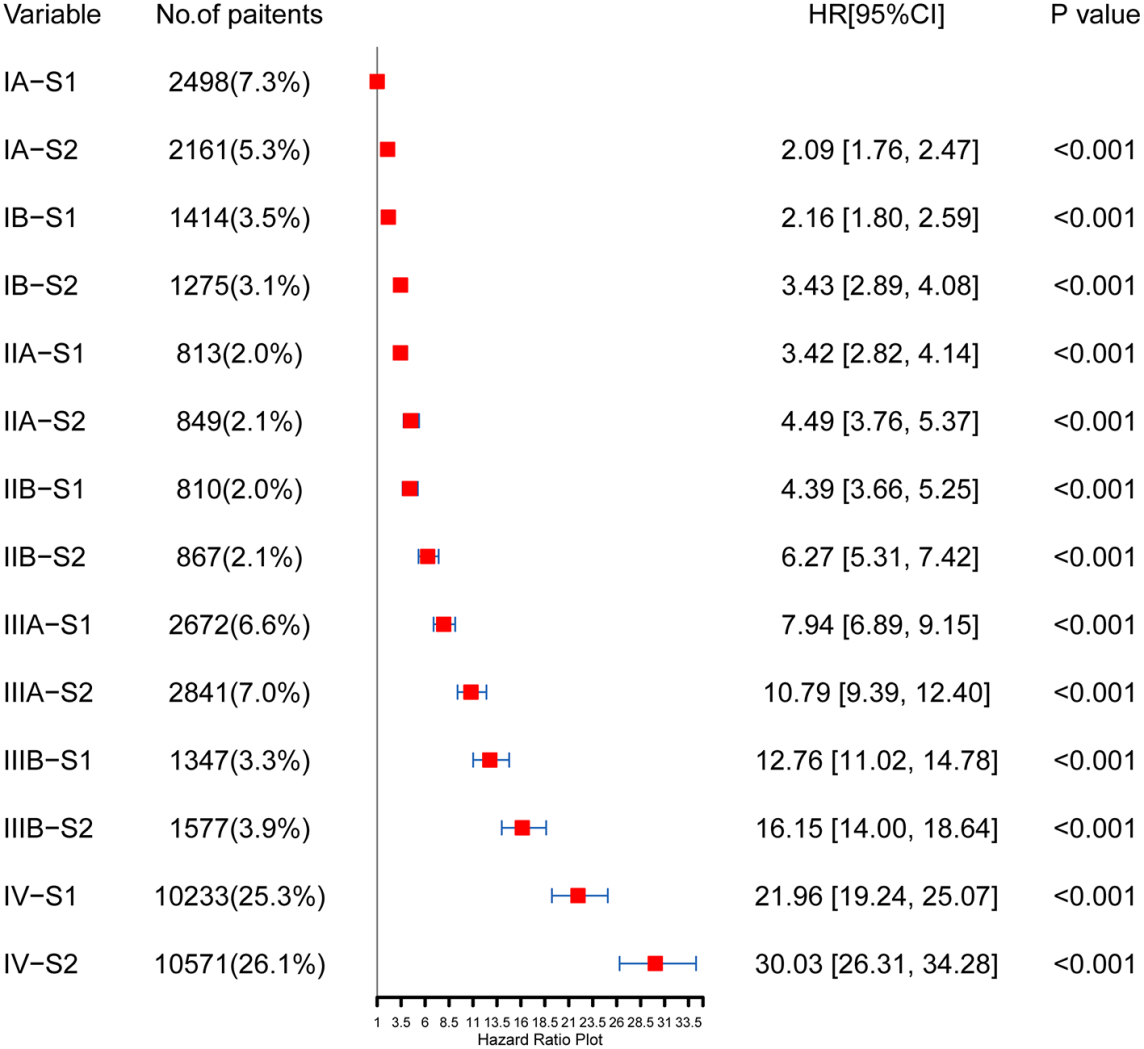
Prognostic value of a novel TNM (tumor node metastasis)-SES (socioeconomic status) stage.

As shown in Figure 4, the HRs of each TNM-SES stage were obtained using the Cox proportional hazards regression model. Consistent with the result of Kaplan–Meier curves, SES-1 stage patients had lower HRs than SES-2 stage patients in each TNM stage. It is worth noting that SES-1 patients had lower HRs than SES-2 patients with advanced TNM stage. For example, IIB-S1 patients [HR = 4.39, 95% CI (3.66–5.25)] had a better prognosis compared to IIA-S2 patients [HR = 4.49, 95% CI (3.76–5.37)].

## Discussion

In our population-based study, we identified four SES factors associated with CSS in patients with NSCLC, which included marital status, insurance status, educational level, and income level, and further constructed a new SES stage based on these factors. Depending on the SES stage, the eligible patients were divided into the SES-1 stage (high SES) and SES-2 stage (low SES). *Our SES stage played an important role in stage at diagnosis, and survival. The higher rate of early TNM stage occurred in SES-1 stage patients than SES-2 patients*. Our results also demonstrated that SES-1 stage patients had a better prognosis than SES-2 patients. Our novel TNM-SES stage, which incorporates the SES stage into the traditional TNM stage, showed a superior predictive performance to the TNM stage.

Four SES factors which were associated with CSS of patients with NSCLC were identified by cox regression analysis, such as insurance status, marital status, country percentage with bachelor's degree and country-level median household income. Then we divided all included patients with NSCLC into two groups based on cutoff SES prognostic score, 20,237 patients in the SES-1 stage and 20,141 patients in the SES-2 stage. Patients in the SES-1 stage have higher SES status than those in the SES-2 stage. Interestingly, there were significant differences (except tumor location) between the two SES stages, such as age, sex, race, histology, site, TNM stage, surgery, and chemotherapy. The proportion of TNM I stage (IA and IB) was higher in patients with SES-1 stage, and patients with SES-2 stage had higher rate of advanced TNM stage (IIIA, IIIB, IV). Our results indicated that advanced NSCLC was associated with low-SES. Hastertet al. (13) demonstrated that people living in the lowest socioeconomic status areas had higher colorectal cancer incidence compared to living in the highest socioeconomic status areas. Another study (14) showed that lower SES was correlated with younger age, Black or Hispanic race/ethnicity, Medicaid/uninsured, and higher T stage. Our results also showed that NSCLC patients with lower SES were associated with young adults, black race, male, squamous carcinoma, upper lobe site and advanced stage. Besides, NSCLC patients with higher SES preferred surgery and chemotherapy, which may explain the better prognosis in those patients.

One prior study (15) with over one million cancer patients demonstrated that married cancer patients were more likely to have metastasis cancer, inconclusive treatment and dismal survival outcome. Several studies (16–19) have shown that unmarried status was a significant poor prognostic factor of survival outcomes in lung cancer and other malignancies. Similar to these findings, our results also showed that patients of never married, divorced and were widowed had a higher risk of death. Some reasons may explain the association between marital status and CSS. Emotional support plays an essential role in the treatment of cancer patients. It was shown (20–22) that unmarried patients experienced more distress and anxiety compared to married patients. Peters et al. (23) found that marital status was positively associated with cancer-specific survival of patients with hepatocellular carcinoma [HR = 0.71, 95% CI (0.55–0.92); *P* < 0.010]. On the other hand, married patients with NSCLC were more likely to regularly receive surveillance and follow-up compared to unmarried patients (24–26). Better obedience may decrease recurrence rate and mortality.

There were several previous reports (27–29) about the association of insurance status and household income with survival outcomes in some malignancies. The disparities of these factors represented the financial ability to deal with the increased medical expenses. Several studies (12, 30, 31) have shown that insurance and high household income were positively associated with a better prognosis for patients with NSCLC, compared to those of Medicaid or uninsured and low household income. Several reasons may explain the impact of insurance status and household income on survival outcomes. Firstly, patients with better financial status were likely at an early stage of disease at presentation or diagnosis (23). In addition, Patients with poor financial status experienced delays in access to regular surveillance and were likely to undergo a lobectomy for early-stage NSCLC (32–34).

It had been reported that education was significantly associated with the incidence and prognosis of lung cancer (35–37). Zhou et al. (38) reported that higher education decreased 52% the risk of lung cancer (OR = 0.48). A study performed by James et al. (39) demonstrated that patients with NSCLC with low education had a worse prognosis than patients with high education. The association between education and the prognosis of patients with NSCLC had not been explanted. Previous studies (40, 41) suggested that education was correlated with income, lifestyle and self-management, which would further impact the survival outcome of cancer patients.

Many previous studies (42, 43) demonstrated that socioeconomic status was significantly associated with the health of the population. SES plays a critical role in several diseases, such as chronic stress, heart disease, ulcers, type 2 diabetes and cancer. In our study, we established a novel SES stage by using four SES factors associated with CSS in patients with NSCLC and further divided the eligible patients into two-stage (SES-1) and SES-2. Our results showed SES-2 stage was significantly associated with CSS of the patients with NSCLC, with a 21.0% increased risk. After adjusting for several confounders, such as histology, race, sex and site, SES-1 stage patients with NSCLC also had a satisfactory prognosis compared to SES-2 stage patients. Patients with the SES-1 stage were more likely to have an early stage of disease at diagnosis and to receive surgery and chemotherapy, which may be associated with the favorable prognosis of patients.

ACJJ staging system was widely performed for stratification to select treatment modality and prognosis prediction in NSLCL patients. However, this TNM stage only incorporated the clinicopathological characteristics of the tumor and not concerned the socioeconomic status. Our results demonstrated SES was significantly associated with the prognosis of patients with NSCLC. The C-index of our novel TNM-SES stage was 0.732, higher than the traditional TNM stage. The higher C-index indicated that TNMS-SES have an excellent value in predicting prognosis. In our results, SES-1 stage patients with NSCLC had a better prognosis than SES-2 stage patients in each TNM stage. It was worth noting that some SES-1 stage patients had a similar prognosis, compared to SES-2 stage patients with a higher TNM stage, which also reflected the superior predictive value of SES.

Reliable results about the association between SES and CSS of patients with NSCLC were attained from our large population-based study. However, there was some limitation in our study. Firstly, we only enrolled five SES factors (marriage, insurance, education, household income and employment) in our study. However, it had been indicated that smoking status was a critical factor associated with CSS in lung cancer patients, which was not available in SEER data. Besides, information in our study was only from the US, which may be applicable in other countries. The impact of SES on CSS of Chinese patients with NSCLC needs to be investigated.

## Conclusion

*Our population-based study aimed to evaluate the impact of SES on cancer staging and survival in NSCLC. Our results demonstrated that NSCLC patients with lower SES were associated with young adult, black race, male, squamous carcinoma, upper lobe site, and advanced stage*. SES was significantly associated with the SCC of patients with NSCLC. Our novel TNM-SES staging system showed a superior predictive value to the traditional TNM stage. The impact of SES on patients with NSCLC should receive more concern in clinical management. *Future efforts should aim to refine and validate our TNM-SES staging system in more clinical centers*.

## Data availability statement

The original contributions presented in the study are included in the article/Supplementary material, further inquiries can be directed to the corresponding authors.

## Ethics statement

The studies involving human participants were reviewed and approved by the Ethics Committee and Institutional Review Board of the Affiliated Changsha Central Hospital. Written informed consent for participation was not required for this study in accordance with the national legislation and the institutional requirements.

## Author contributions

XY: methodology, investigation, resources, and writing—original draft. GW: conceptualization, supervision, and writing—review and editing. LD: software and formal analysis. ML: validation and resources. YZ: formal analysis, project administration, and visualization. All authors contributed to the article and approved the submitted version.

## Funding

This work was supported through the project of Municipal Heath Committee of Hunan Province (No. 20201372) and Chinese Medicine research project of Hunan Province (No. 2019135).

## Conflict of interest

The authors declare that the research was conducted in the absence of any commercial or financial relationships that could be construed as a potential conflict of interest.

## Publisher's note

All claims expressed in this article are solely those of the authors and do not necessarily represent those of their affiliated organizations, or those of the publisher, the editors and the reviewers. Any product that may be evaluated in this article, or claim that may be made by its manufacturer, is not guaranteed or endorsed by the publisher.

## Supplementary material

Supplementary Figure 1 Kaplan-Meier curves showed patients with the SES-1 stage had a better prognosis than patients with the SES-2 stage in the stratified analysis by histology, race, sex, and site.
