# Supplementary figures and images for: Impact of socioeconomic status on cancer staging, survival in non-small cell lung cancer
Source: Front Public Health. 2022 Nov 8;10:992944. doi: 10.3389/fpubh.2022.992944 (PMC9679653; doi:10.3389/fpubh.2022.992944)

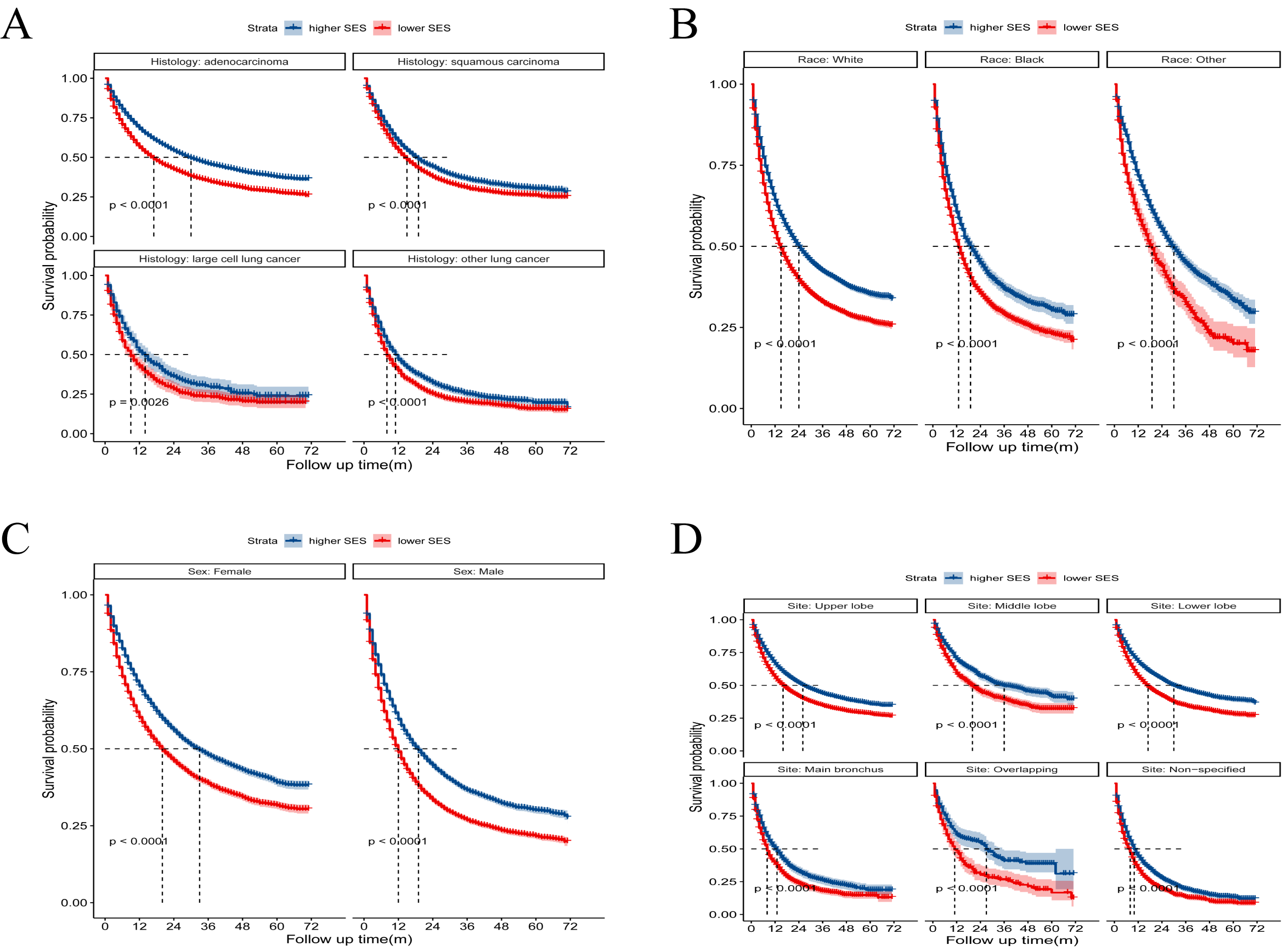

Supplement: Supplementary Figure 1 — Kaplan-Meier curves showed patients with the SES-1 stage had a better prognosis than patients with the SES-2 stage in the stratified analysis by histology, race, sex, and site. [file Image_1.TIF]
